# Data on farm diversification decisions and farmers’ risk preferences in the Ruhr Metropolitan region (Germany)

**DOI:** 10.1016/j.dib.2018.03.008

**Published:** 2018-03-07

**Authors:** Manuela Meraner, Bernd Pölling, Robert Finger

**Affiliations:** a Agricultural Economics and Policy Group, ETH Zurich, Zurich, Switzerland; b Institute for Food and Resource Economics, University of Bonn, Bonn, Germany; c South-Westphalia University of Applied Sciences, Department of Agriculture, Soest, Germany

## Abstract

On-farm non-agricultural diversification is increasingly important for many farmers to stabilize their income and to fulfill societal demand for integrated farming. Especially in peri-urban areas, where the potential for farms’ expansion in terms of farm size is limited and public interest in regional products and other services provided at farms is high, the uptake of on-farm non-agricultural diversification is thus an attractive strategy for many farmers. The here presented dataset is based on an online survey conducted in March 2016 with 156 farms located in the Ruhr Metropolitan region (North Rhine-Westphalia, Germany). The survey was used to collect information on farms’ and farmers’ characteristics. More specifically, the dataset includes detailed information on farms’ diversification decisions, location, size and general information on farms’ production type and structure. Moreover, the dataset contains information on farmers’ risk perception and risk preferences (collected using three different elicitation methods) and general information on farmers and households. In addition, farmers’ personal assessments on agricultural production in peri-urban settings and information on the use of extension services were collected.

**Specifications table**

**Table:** 

Subject area	*Agricultural Economics, Experimental Economics, Agriculture*
More specific subject area	*Risk preference elicitation, risk perception, diversification, peri-urban agriculture*
Type of data	*CSV File, text file*
How data was acquired	*Online Survey*
Data format	*Raw and partially analyzed data*
Experimental factors	*n.a.*
Experimental features	*n.a.*
Data source location	Ruhr Metropolitan region (Germany)
Data accessibility	Data provided in the article is accessible to the public

**Value of the data**

• The data contains information on farm-diversification, farm and farmers characteristics that could be used by other researchers.
• The data allows for comparison of farmers’ risk preferences with other studies and can be used in meta-analysis.
• The data allows analysis and comparison of farm diversification decisions and their determinants.
• The data reveals insights in farm-level production and farm-diversification decision in peri-urban agriculture.

## 1 Data

The data includes results from an online survey sample of 156 farmers located in the peri-urban area of the Ruhr Metropolitan region. The dataset includes information on the farmers risk perception and detailed information on the farms’ diversification activities as well as other farm, farmer and household characteristics as well as results of three different risk preference elicitation methods. The dataset, the survey as well as further details to the risk preference elicitation tasks are made available along with this article.

## 2 Experimental design, materials and methods

An online survey was conducted with farmers in the Ruhr Metropolitan region in March 2016. The survey was distributed via email through the Chamber of Agriculture to in total 2368 farm managers, representing about 70% of the farms situated in Ruhr Metropolitan region [1]. The email included a direct link to a web survey site that was created with the software LimeSurvey. An email reminder was sent to the farmers two weeks after the first contact. The survey was tested in 17 iterative pre-tests with farm managers and agricultural students located outside Ruhr Metropolitan region. Almost all items were constructed as closed questions to provide simple usage of the web survey tool. However, farmers could add additional comments for the majority of questions. The overall response rate was 14% and half of these respondents fully completed the survey, leading to a sample size of 156 farmers. The survey included questions on (see all questions in the Appendix 2 and App link to 3):

(i) the farm characteristics (e.g. production type, full- or part-time farm, and cultivated area),
(ii) the location of the farm (e.g. perception of rural-urban relations, self-assessment of the farms location, assessment of advantages and disadvantages of an urban location),
(iii) the use of extension service (e.g. used consultancy over the last five years),
(iv) marketing and diversification decisions (e.g. used marketing channels, adopted diversification activities),
(v) farmers’ risk perception, self-assessed risk attitude (e.g. [2], [3]) as well as self-assessment of severe losses within the past five years
(vi) risk preference elicitation based on a contextualized lottery over 10 questions on willingness to take risks [4],
(vii) characteristics of the farm manager and the household as well as additional workforce (e.g. age of the farm manager, household size, additional workforce, share of total workforce in agriculture and diversification activities), and
(viii) personal future outlook of the respondents’ farms (e.g. future plans for the agricultural and diversification business).

Furthermore, the time needed for each part of the survey was recorded. Additionally, publicly available geo data were added to the web survey's primary database (note that information on the exact location is removed from the here published data to guarantee anonymity of the respondents). This enabled us to connect the survey information with data on soil fertility and distance to urban centers for 132 of the 156 farms.

Note that all of the data was collected anonymously and voluntary, all participants where made aware of the anonymized data processing procedure after the end of the data collection.

### 2.1 Eliciting risk perception

To measure the farmers’ subjective risk perception we asked farmers to score the perceived probability of five different risk sources on a five point Likert scale from 1 (very unlikely) to 5 (very likely) and the perceived impact for each source on a five point Likert scale from 1 (very small impact) to 5 (very big impact). The perceived risk scores are calculated by multiplying the perceived probability of occurrence with the perceived impact [2], [3], [4]. The risk sources included in the survey where based on the in-depth expert interviews (see [5]) as well as a literature study [3], [4], [6]. Namely the risk sources included in the survey are i) market and price risks (e.g. price volatility), ii) political and structural risks (e.g. decreasing direct payments), production risks (e.g. yield volatility), financial risks (e.g. liquidity shortage) and other risks (e.g. shortfall of qualified workforce).

### 2.2 Eliciting risk preferences

The dataset includes in total three different methods to elicit risk preference from the respondents. First, we included a straightforward self-assessment of the willingness to take risk: ‘On a scale from 0 to 10, where 0 means ‘not at all willing to take risks’ and 10 means ‘very willing to take risks’, how would you assess your personal preference to take risks?’ (following Dohmen et al. [2]). Second, we used the following four business statements related to three major sources farmers are exposed to and to agriculture in general: On a scale from 1 to 5, where 1 means ‘fully agree’ and 5 means ‘don't agree’ please indicate your position on the following statements: ‘I am willing to take more risks than my colleagues with respect to (1) … production risks; (2) … marketing and pricing risks; (3) … financial risks; (4) … farming in general’. These contextualized questions follow other studies [2], [3], [7] ensuring comparability of results. The third measure of risk preferences elicited from the sample of farmers was a contextualized multiple price list following Musser and Patrick [8]. Farmers were asked to choose between two options (A or B) for ten different situations with varying probabilities. A risk-neutral person would select option A in the first four rows and option B in the last six rows. We create a realistic payout structure of the contextualized MPL using payouts ranging from 5€ to 192.5€. The wording used in the lottery was explicitly framed in an agricultural context. Furthermore, we reduce complexity and consequential inconsistent behavior by including a pie chart displaying proportions next to the verbal presentation of decisions as a visual aid [9], [10], [11]. Instructions and an example of the visual representation can be found in Appendix 1). To incentivize the MPL we chose between-subjects random incentive system following [12], [13]. More specifically, 10% of all participants are selected at the end of the survey period for real payouts based on their choices. Farmers were informed about that procedure before starting the survey and payouts were distributed via bank transfer one month after the first contact.

## Appendix A Supplementary material

Supplementary material Appendix 1: Instructions to contextualized multiple price list.

Supplementary material Appendix 2: Variable coding and definitions.

Supplementary material Appendix 3: The survey (in German).

Supplementary material Appendix 4: the dataset (as csv file).

## Footnotes

Appendix A Supplementary data associated with this article can be found in the online version at doi:10.1016/j.dib.2018.03.008.
