# Supplementary material for: Data on farm diversification decisions and farmers’ risk preferences in the Ruhr Metropolitan region (Germany)
Source: Data Brief. 2018 Mar 7;18:9–12. doi: 10.1016/j.dib.2018.03.008 (PMC5996160; doi:10.1016/j.dib.2018.03.008)
Supplement: Supplementary file 2 — Supplementary material Appendix 1: Instructions to contextualized multiple price list. [file mmc2.docx]

**Appendix 1**

**Instructions to contextualized** multiple price list (translated from German):

To make sure that you understand the payout structure regarding your participation in this survey, please read the following instructions carefully:

Below you see a table including 10 different decision scenarios (rows) for possible investment outcomes (A and B). Each row of the decision table contains a pair of choices between Option A and Option B. 10 out of 100 participants will be chosen randomly as winners. If you are one of them your payout will be calculated as following:

1) One of the rows is selected at random, and the Option (A or B) that you chose in that row will be used to determine your earnings.

Example: We assume row 1 was selected randomly and your selected choice is investment A.

2) After one of the decisions has been randomly selected, another random number is chosen to elicit the probability of your payout. This random number determines your earnings for the Option (A or B) that you previously selected for the decision being used.

Example: In row 1 we randomly make a selection out of 10 balls (1 green and 9 blue) to determine your payout. If a blue ball is selected, the amount is 80.000€. The actual payout is divided by 1.000. Thus, you receive a payout of 80€.


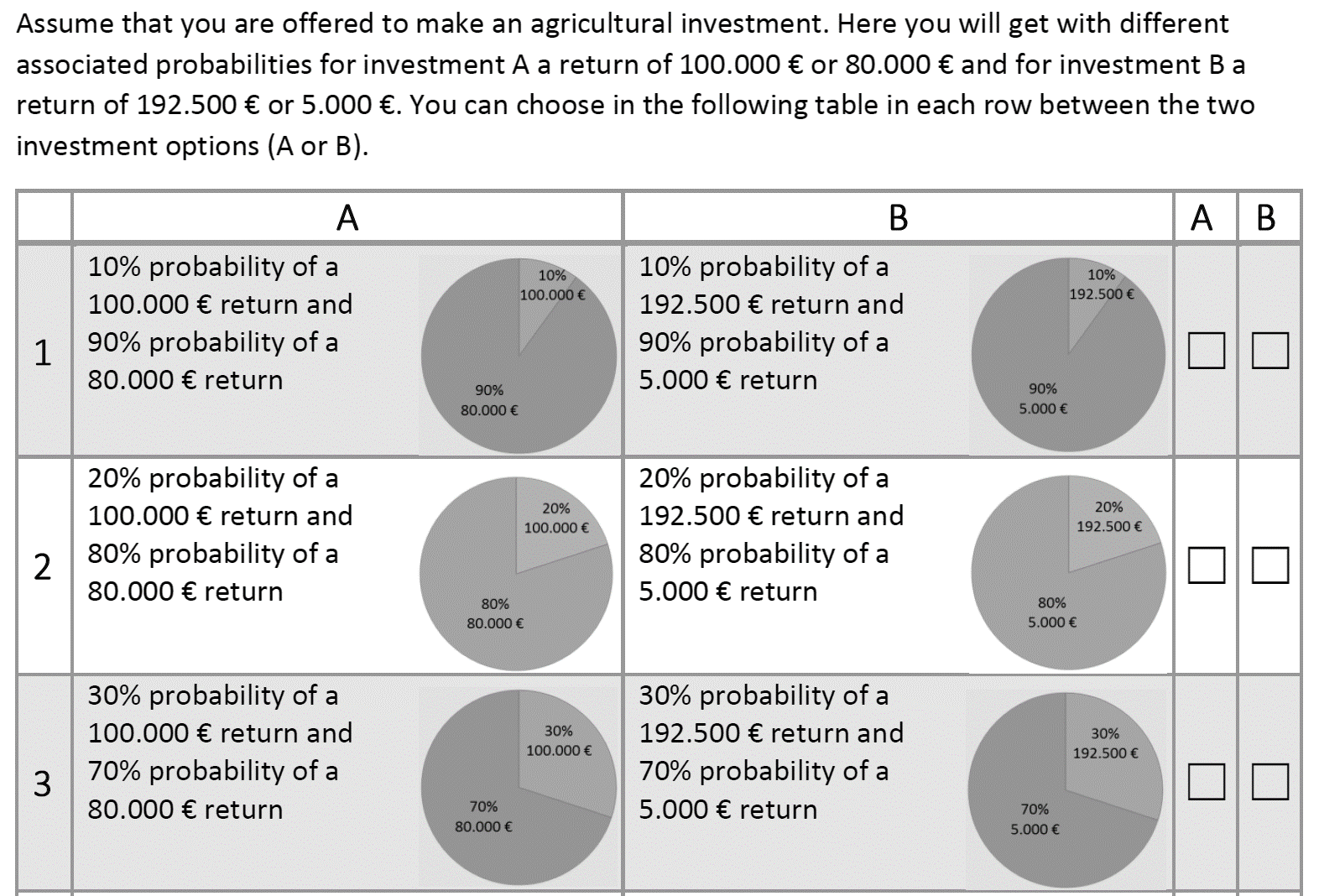


Fig A1 Example visual presentation MPL (from Meraner and Finger, Data on German farmers risk preference, perception and management strategies, Data in brief, 15 (2017) 102-105.)
