# Supplementary material for: Data on farm diversification decisions and farmers’ risk preferences in the Ruhr Metropolitan region (Germany)
Source: Data Brief. 2018 Mar 7;18:9–12. doi: 10.1016/j.dib.2018.03.008 (PMC5996160; doi:10.1016/j.dib.2018.03.008)
Supplement: Supplementary file 3 — Supplementary material Appendix 2: Variable coding and definitions. [file mmc3.docx]

**Appendix 2: Variable coding and definitions**

| Question in German | Question in English | Variable name | Scaling |
| --- | --- | --- | --- |
| A: Betriebliche Informationen | A: Operational information |  |  |
| 1. Betreiben Sie Ihren Betrieb im Haupt- oder Nebenerwerb? | 1. Are you working your farm as a full- or part-time farmer? | Haupt-Nebenerwerb | 1= full time farmer  0= part time farmer |
| 2. Wirtschaften Sie konventionell oder nach ökologischen Richtlinien? | 2. Is your production organic or conventional? | Bio | 1= organic farm  0= non-organic farm |
| 3. Wie ist der aktuelle Stand Ihrer Flächenausstattung? | 3. How is your total area divided into? |  |  |
| 3.1 Landwirtschaftlich genutzte Fläche | 3.1 agricultural land | Flaeche_landw_total | ha of agricultural land |
| 3.2 Ackerland | 3.2 arable area | Flaeche_acker | ha of arable land |
| 3.3 Grünland | 3.3 grassland | Flaeche_gruenland | ha of grassland |
| 4. Wie viel Hektar haben Sie derzeit gepachtet? | 4. What is the size of your rented land? | Pachtflaeche | ha of rented land |
| 5. Welche Kulturen bauen Sie derzeit in welchem Umfang an? | 5. What kind of crops do you have to which extent? |  |  |
| 5.1 Getreide | 5.1 grain | Getreide | ha of grain area |
| 5.2 Hackfrüchte | 5.2 root crop | Hackfruechte | ha of root crop area |
| 5.3 Sonderkulturen | 5.3 horticulture | Sonderkulturen | ha of horticulture |
| 5.4 Dauerkulturen | 5.4 permanent crop | Dauerkulturen | ha of permanent crop area |
| 5.5 Futterpflanzen | 5.5 forage | Futterpflanzen | ha of forage area |
| 5.6 Hülsenfrüchte | 5.6 legume | Huelsenfruechte | ha of legume area |
| 5.7 Ölsaaten | 5.7 oilseed | Oelsaaten | ha of oilseed area |
| 5.8 Grünland | 5.8 grassland | Grassland area | ha of grassland area |
| 5.9 Sonstige | 5.9 other crops | Sonst_Ackerkulturen | ha of other area |
| 6. Bauen Sie Kulturen in Gewächshäusern und unter Folien an? | 6. Do you have cultures in greenhouses or foil tunnels? |  | (select one or more options) |
| 6.1Gewächshaus | 6.1 greenhouse | Gewaechshaus | 1= yes |
| 6.2 Folientunnel | 6.2 foil tunnel | Folie | 1= yes |
| 6.3 Keine Gewächshäuser und Folientunnels | 6.3 neither greenhouses nor foil tunnel | kein_Gewaechshaus_o_Folie | 1= yes |
| 6.4 Falls ja, wie viele m^2^ in Gewächshäusern? | 6.1 If yes, what is the area of greenhouses? | Flaeche_unter_glas | m^2^ of greenhouse area |
| 6.5 Falls ja, wie viele m^2^ unter Folientunnels? | 6.2 If yes, what is the area of foil tunnels? | Flaeche_unter_folie | m^2^ of foil tunnels |
| 7. Wie viele Tiere halten Sie auf dem Betrieb? | 7. What is the amount of livestock on your farm? |  |  |
| 7.1 Mutterkühe | 7.1 mother cow | Mutterkuehe | nr of mother cows |
| 7.2 Fleischrinder/ Bullen | 7.2 cattle | Fleischrinder_Bullen | nr of cattle |
| 7.3 Sauen | 7.3 sow | Sauen | nr of sow |
| 7.4 Schweinemast | 7.4 fattening pigs | Schweinemast | nr of fattening pigs |
| 7.5 Legehennen | 7.5 laying hens | Legehennen | nr of laying hens |
| 7.6 Geflügelmast | 7.6 fattening poultry | Gefluegelmast | nr of fattening poultry |
| 7.7 Schafe/ Ziegen | 7.6 sheep/ goat | Schafe_Ziegen | nr of sheep and goat |
| 7.8 Pferde | 7.7 horses | Pferde | nr of horses |
| 7.9 Sonstiges | 7.8 other animals | Sonst_Tiere | nr of other animal |
| B: Lagebezug | B: location |  |  |
| 8. Wie stark unterscheiden sich Ihrer Meinung nach die städtische und ländliche Landwirtschaft? | 8. How strong is the difference between urban and rural agriculture? | Unterschied_Stadt_laendl_Landwirtsch | scale from 1 to 100  0 = no difference  100= very big difference |
| 9. Liegt Ihr Betrieb in einer städtischen oder ländlichen Umgebung? | 9. Is your farm situated rather urban or rural? | Lage_des_Betriebs | scale from 1 to 100  0= very rural  100= very urban |
| 10. Bietet das städtische Umfeld für Ihre Betriebsausrichtung mehrheitlich Vor- oder Nachteile? | 10. Does an urban environment provide rather advantages or rather disadvantages to your farm? | Stadt_Vor-Nachteile | scale from 1 to 100  0= only advantages  100= only disadvantages |
| 11. Welche Vorteile eines städtischen Umfelds fallen Ihnen für Ihre Betriebsausrichtung ein? | 11. What advantages for your type of farm can you think of regarding an urban environment? |  | (select one or more options) |
| 11.1 Großer Verbrauchermarkt in der Region | 11.1 lots of potential customers in the region | Vort_Verbrauchernaehe | 1= yes |
| 11.2 Kaufkraft in der Region | 11.2 purchase power in the region | Vort_Kaufkraft | 1= yes |
| 11.3 Gute Infrastruktur (Verkehr, Medien) | 11.3 infrastructure (traffic, media) | Vort_Infrastruktur | 1= yes |
| 11.4 Vor- und nachgelagerte Industrie in der Region | 11.4 supply chain in the region | Vort_Industrie | 1= yes |
| 11.5 Urbane Ressourcen nutzbar (Wärme, organische Abfälle, etc.) | 11.5 urban resources useable | Vort_Urbane_Ressourcen_nutzbar | 1= yes |
| 11.6 Sonstiges und zwar: | 11.6 other advantages: | Sonst_Vort_Stadt | open question |
| 11.7 Keine | 11.7 no advantages | Vort_ Keine | 1= yes |
| 12. Welche Nachteile eines städtischen Umfelds fallen Ihnen für Ihre Betriebsausrichtung ein? | 12. What disadvantages for your type of farm can you think of regarding an urban environment? |  |  |
| 12.1 Land: Flächenknappheit und weiterer Flächenverlust | 12.1 land: scarcity of land and further loss of land | Nacht_Land_Flaechenknappheit | 1= yes |
| 12.2 Land: Flächenzersplitterung | 12.2 land: space fragmentation | Nacht_Land_Flaechenzersplitterung | 1= yes |
| 12.3 Hoher Pachtflächenanteil mit kurzen Laufzeiten | 12.3 lots of short–term rented land | Nacht_Pacht | 1= yes |
| 12.4 Akzeptanzprobleme, kritische Nachbarschaft | 12.4 problems of acceptance/ critical neighborhood | Nacht_Akzept | 1= yes |
| 12.5 Vandalismus, Diebstahl | 12.5 vandalism, theft | Nacht_Vandalismus | 1= yes |
| 12.6 Keine Nachteile | 12.6 no disadvantages | keine_Nacht | 1= yes |
| 12.7 Sonstiges, und zwar: | 12.7 other disadvantages: | Sonst_Nacht | open question |
| 13. Bitte geben Sie Ihre Postleitzahl an: | 13. What is your postal code? | PLZ | postal code of farm location |
| C: Beratung | C: consultation |  |  |
| 14. Nutzen Sie derzeit oder haben Sie in den letzten 5 Jahren Beratungsangebote in Anspruch genommen? | 14. Have you used any consultations in the past 5 years? | Beratung | 1= yes  0= no |
| 15. (Wenn Frage 14 ja) Welche Beratungsangebote der LWK haben Sie in den letzten 5 Jahren in Anspruch genommen? | 15. (If question A yes) What kind of consultations provided by the extension service have you experienced in the past 5 years? |  |  |
| 15.1 Beratung zur Unternehmensführung | 15.1 consultation on corporate governance | Ber_Unternehmen | 1= yes  0= no |
| 15.2 Beratung zu Förderungsmaßnahmen | 15.2 consultation on subsidy/ direct payment | Ber_Foerderung | 1= yes  0= no |
| 15.3 Beratung im Ackerbau | 15.3 consultation on plant production | Ber_Acker | 1= yes  0= no |
| 15.4 Beratung zur Rinderhaltung | 15.4 consultation on cattle husbandry | Ber_Rind | 1= yes  0= no |
| 15.5 Beratung zur Schweinehaltung | 15.5 consultation on pig husbandry | Ber_Schwe | 1= yes  0= no |
| 15.6 Beratung zur Geflügelhaltung | 15.6 consultation on chicken husbandry | Ber_Gefl | 1= yes  0= no |
| 15.7 Beratung zur Pferdehaltung | 15.7 consultation on horse keeping | Ber_Pfer | 1= yes  0= no |
| 15.8 Beratung zur Schafhaltung | 15.8 consultation on sheep husbandry | Ber_Schaf | 1= yes  0= no |
| 15.9 Beratung im Ökologischen Landbau | 15.9 consultation on organic farming | Ber_Bio | 1= yes  0= no |
| 15.10 „Landservice“ Beratung (Diversifizierungsangebote) | 15.10 consultation by “Landservice” (possibilities of diversification) | Ber_Divers | 1= yes  0= no |
| 15.11 Beratung im Gartenbau | 15.11 consultation on horticulture | Ber_Gart | 1= yes  0= no |
| 15.12 Beratung Wasserrahmenrichtlinie | 15.12 consultation on water framework directives | Ber_Wasser | 1= yes  0= no |
| 15.13 Beratung zum Klimaschutz | 15.13 consultation on climate protection | Ber_Klima | 1= yes  0= no |
| 15.14 Sonstige Beratung: | 15.14 other consultations: | Sonst_Ber | open question |
| 16. Wäre Ihrer Meinung nach eine Beratung speziell für Landwirtschaftsbetriebe im städtischen Umfeld geeignet oder nicht? | 16. Do you think that a special consultation for farms within an urban environment would be beneficial? | Ber_Stadt | scale from 1 to 100  0= no benefits at all  100= very beneficial |
| 17. Wäre Ihrer Meinung nach eine Beratungsregion “Metropole Ruhr” geeignet oder nicht? | 17. Do you think that a consultation region „Ruhr Metropolitan region“ would be suitable? | Ber_Ruhrregion | scale from 1 to 100  0= very unsuitable  100= very suitable |
| D: Vermarktung und Diversifizierung | D: Marketing and diversification |  |  |
| 18. Welche Art von Einkommenskombinationen nutzen Sie in Ihrem Betrieb? | 18. What sources of income do you have on your farm? |  |  |
| 18.1 Tourismus | 18.1 tourism |  |  |
| 18.1.1 Gastronomie | 18.1.1 gastronomy | Tour_Gastro | 1= yes |
| 18.1.2 Urlaub (Beherbergung) | 18.1.2 Accomodation (lodging) | Tour_Beherbergung | 1= yes |
| 18.1.3 Vermietung von Räumlichkeiten für Ferien und Tagungen | 18.1.3 rent of facilities for vacation and conferences | Tour_Vermietung | 1= yes |
| 18.1.4. Pferdehof/ Pensionspferde | 18.1.4 equestrian services | Tour_Pferdehof | 1= yes |
| 18.1.5 Veranstaltungen, Sport- oder Freizeitaktivitäten (Spielangebote für Kinder, Führungen, Fahrrad- und Kanuverleih etc.) | 18.1.5 events/ sport and leisure activities (games for children, guided tours, rent of bicycles and canoes) | Tour_Freizeit | 1= yes |
| 18.1.6 Sonstiges und zwar: | 18.1.6 other tourism activities: | Tour_sonst | 1= yes |
| 18.2. Bitte geben Sie für die angekreuzten Aktivitäten an, seit welchem Jahr Sie diese betreiben. | 18.2 Since when do you engage in these activities? |  |  |
| 18.2.1 Gastronomie | 18.2.1 gastronomy since | Tour_Gastro_seit | start year of gastronomy |
| 18.2.2 Urlaub (Beherbergung) | 18.2.2 accomodation (lodging) since | Tour_Beherbergung_seit | start year of accomodation |
| 18.2.3 Vermietung von Räumlichkeiten für Ferien und Tagungen | 18.2.3 rent of facilities for vacation and conferences since | Tour_Vermietung_seit | start year of rent of facilities |
| 18.2.4 Pferdehof, Pensionspferde | 18.2.4 equestrian services since | Tour_Pferdehof_seit | start year of equestrian services |
| 18.2.5 Veranstaltungen, Sport- oder Freizeitaktivitäten (Spielangebote für Kinder, Führungen, Fahrrad- und Kanuverleih etc.) | 18.2.5 events/ sport and leisure activities (games for children, guided tours, rent of bicycles and canoes) since | Tour_Freizeit_seit | start year of events and leisure activities |
| 18.2.6 Sonstige Tourismus Aktivitäten, seit | 18.2.6 other tourism activities since | Tour_sonst_seit | start year of other tourism activities |
| 18.2.7 Sonstiges und zwar: | 18.2.7 other tourism activities: | Tourismus_Sonstiges | open question |
| 18.3 Dienstleistungen | 18.3 services |  |  |
| 18.3.1 Soziale Dienstleistungen | 18.3.1 social services | Dienstl_sozial | 1= yes |
| 18.3.2 Land-/ Forst-/ Hauswirtschaftliche Dienstleistungen (kommunale und private) | 18.3.2 land-/ forest- and household services (public and private) | Dientsl_landw | 1= yes |
| 18.3.3 Sonstiges, und zwar: | 18.3.3 other services | Dienstl_sonst | 1= yes |
| 18.4 Bitte geben Sie für die angekreuzten Aktivitäten an, seit welchem Jahr Sie diese betreiben. | 18.4 Since when do you engage in these activities? |  |  |
| 18.4.1 Soziale Dienstleistungen | 18.4.1 social services since | Dienstl_sozial_seit | start year of social service |
| 18.4.2 Land-/ Forst-/ Hauswirtschaftliche Dienstleistungen (kommunale und private) | 18.4.2 land-/ forest- and household services (public and private) since | Dientsl_landw_seit | start year of land-/ forest and household service |
| 18.4.3 Sonstige Dienstleistungen, seit | 18.4.3 other services since | Dienstl_sonst_seit | start year of other service |
| 18.4.4 Sonstiges, und zwar: | 18.4.4 other services: | Dienstleistung_Sonstiges | Open question |
| 18.5 Erneuerbare Energien | 18.5 renewable energy |  |  |
| 18.5.1 Nachwachsende Rohstoffe (Biomasse) | 18.5.1 renewable raw materials (biomass) | Energie_nachw | 1= yes |
| 18.5.2 Nicht- nachwachsende Rohstoffe (Sonne, Wind, Erdwärme, etc.) | 18.5.2 nonrenewable raw materials (radiation, wind, geothermal energy, etc.) | Energie_nicht_nachw | 1= yes |
| 18.5.3 Weitere | 18.5.3 other energy gaining activities | Energie_sonst | 1= yes |
| 18.6 Bitte geben Sie für die angekreuzten Aktivitäten an, seit welchem Jahr Sie diese betreiben. | 18.6 Since when do you engage in these activities? |  |  |
| 18.6.1 Nachwachsende Rohstoffe (Biomasse) | 18.6.1 renewable raw materials (biomass) since | Energie_nachw_seit | start year of renewable raw materials |
| 18.6.2 Nicht-nachwachsende Rohstoffe (Sonne, Wind, Erdwärme, etc.) | 18.6.2 non-renewable raw materials (radiation, wind, geothermal energy, etc.) since | Energie_nicht_nachw_seit | start year of non-renewable raw materials |
| 18.6.3 Weitere Energie seit | 18.6.3 other energy gaining activities: since | Energie_sonst_seit | start year of other energy gaining activities |
| 18.6.4 Weitere | 18.6.4 other energy gaining activities: since | Energie_Sonstige | open question |
| 18.7 Weiterverarbeitung landwirtschaftlicher Produkte | 18.7 Processing of agricultural products |  |  |
| 18.7.1 Weiterverarbeitung pflanzlicher Produkte | 18.7.1 Processing of plant products | Weiterverarb_pfl | 1= yes |
| 18.7.2 Weiterverarbeitung tierischer Produkte | 18.7.2 Processing of animal products | Weiterverarb_tier | 1= yes |
| 18.8.1 Verkaufen Sie die weiterverarbeiteten pflanzlichen/ tierischen Produkte direkt? | 18.8.1 Do you sell the processed products directly? |  |  |
| 18.8.2 Verkaufen Sie die weiterverarbeiteten pflanzlichen Produkte direkt? | 18.8.2 Do you sell the processed plant products directly? | Weiterverarb_pfl_direktverk | 1= only direct marketing  0.5 = part of it  0= no |
| 18.8.3 Verkaufen Sie die weiterverarbeiteten tierischen Produkte direkt? | 18.8.3 Do you sell the processed animal products directly? | Weiterverarb_tier_direktverk | 1= only direct marketing  0.5 = part of it  0= no |
| 18.9 Bitte geben Sie für die angekreuzten Aktivitäten an, seit welchem Jahr Sie diese betreiben. | 18.9 Since when do you engage in these activities? |  |  |
| 18.9.1 Pflanzliche Produkte | 18.9 plant products since | Weiterverarb_pfl_seit | start year of processed plant products |
| 18.9.2 Tierische Produkte | 18.9 animal products since | Weiterverarb_tier_seit | start year of processed animal products |
| 18.10 Direktvermarktung (Hofladen, Verkaufswagen) | 18.10 direct marketing |  |  |
| 18.10.1 Hofladenverkauf | 18.10.1 on-farm shop | Direkt_Hofl | 1= yes |
| 18.10.2 Verkaufswagen (Lieferdienst) | 18.10.2 delivery service | Direkt_Verkaufswagen | 1= yes |
| 18.10.3 Verkaufsstand (Bauern- / Wochenmarkt) | 18.10.3 sales booth (market) | Direkt_Markt | 1= yes |
| 18.10.4 Verkaufsstand (Straße) | 18.10.4 sales booth (street) | Direkt_Strasse | 1= yes |
| 18.10.5 Verkaufsautomat | 18.10.5 vending machine | Direkt_Automat | 1= yes |
| 18.10.6 Party service | 18.10.6 party-service | Direkt_Part1 | 1= yes |
| 18.10.7 Sonstiges | 18.10.7 other direct marketing | Direkt_sonst | 1= yes |
| 18.11 Bitte geben Sie für die angekreuzten Aktivitäten an, seit welchem Jahr Sie diese betreiben. | 18.11 Since when do you engage in these activities? |  |  |
| 18.11.1 Hofladenverkauf | 18.11.1 on-farm shop since | Direkt_Hofl_seit | start year of on-farm shop |
| 18.11.2 Verkaufswagen (Lieferdienst) | 18.11.2 delivery service since | Direkt_Verkaufswagen_seit | start year of delivery service |
| 18.11.3 Verkaufsstand (Bauern- / Wochenmarkt) | 18.11.3 sales booth (market) since | Direkt_Markt_seit | start year of sales booth (market) |
| 18.11.4 Verkaufsstand (Straße) | 18.11.4 sales booth (street) since | Direkt_Strasse_seit | start year of sales booth (street) |
| 18.11.5 Verkaufsautomat | 18.11.5 vending machine since | Direkt_Automat_seit | start year of vending machine |
| 18.11.6 Party service | 18.11.6 party-service since | Direkt_Party_seit | start year of party service |
| 18.11.7 Andere | 18.11.7 other direct marketing activities: since | Direkt_sonst_seit | start year of other direct marketing activities |
| 18.11.8 Andere | 18.11.8 other direct marketing activities: | Direktvermarktung_Sonstige | open question |
| 19.1 Mit ca. welchen Anteilen vermarkten Sie Ihre Produkte direkt? | 19.1 What is the share of products sold directly? | Anteil_direktverm | share of products sold directly |
| 19.2 Mit ca. welchen Anteilen vermarkten Sie Ihre Produkte indirekt? | 19.2 What is the share of products sold not directly? | Anteil_indirektverm | share of products sold not directly |
| 20. Welche Kanäle verwenden Sie für Ihre indirekte Produktvermarktung? | 20. What kind of distribution channels do you have for indirect marketing? |  |  |
| 20.1 Großmarkt | 20.1 warehouse | Indirektverm_Grosshandel | 1= yes |
| 20.2 Genossenschaften | 20.2 cooperative | Indirektverm_Genoss | 1= yes |
| 20.3 Regionaler Einzelhandel (Supermarkt, Discounter) | 20.3 regional retailer | Indirektverm_Einzelh | 1= yes |
| 20.4 Weiterverarbeitender Zwischenhändler (Metzger, Bäcker, Gastronomie) | 20.4 processors (butcher, baker, gastronomy) | Indirektverm_Zwischenhaendler | 1= yes |
| 20.5 Erzeugergemeinschaft | 20.5 producer association | Indirektverm_Erzeugergem | 1= yes |
| 20.6 Sonstige | 20.6 other distribution channels: | Indirektverm_sonst | open question |
| 21.1 Sonstige Einkommenskombination | 21.1 other diversification measures: | EKK_Sonstige | open question |
| 21.2 Bitte geben Sie für die sonstigen Einkommenskombinationen an, seit welchem Jahr Sie diese betreiben. | 21.2 Since when do you engage in these diversification measures? | EKK_Sonstige_seit | start year of other diversification measures |
| 22. Keine Einkommenskombination | 22. no diversification measures | EKK_keine | 1= yes |
| 23. Welche Kanäle verwenden Sie für Ihre Produktvermarktung? | 23. What distribution channels do you use for marketing? |  |  |
| 23.1 Großhandel | 23.1 warehouse | Produktverm_Grosshandel | 1= yes |
| 23.2 Genossenschaften | 23.2 cooperative | Produktverm_Genoss | 1= yes |
| 23.3 Regionaler Einzelhandel | 23.3 regional retailer | Produktverm_Einzelh | 1= yes |
| 23.4 Weiterverarbeitender Zwischenhändler (Metzger, Bäcker, Gastronomie, etc.) | 23.4 processors (butcher, baker, gastronomy) | Produktverm_Zwischenhaendler | 1= yes |
| 23.5 Erzeugergemeinschaft | 23.5 producer association | Produktverm_Erzeugergem | 1= yes |
| 23.6 Sonstiges, und zwar: | 23.6 other distribution channel for marketing: | Produktverm_sonst | open question |
| 24. Können bei Ihnen Kunden aktiv in der Produktion bzw. im Management aktiv partizipieren/ teilhaben? | 24. Can customers participate actively in production or management? |  |  |
| 24.1 Ja, Selbstpflückfelder | 24.1 yes, pick your own fields | Mitarb_Selbstpfl | 1= yes |
| 24.2 Ja, Mietgärten | 24.2 yes, gardens for rent | Mitarb_Mietg | 1= yes |
| 24.3 Ja, Solidarische Landwirtschaft | 24.3 yes, Community Supported Agriculture | Mitarb_Solawi | 1= yes |
| 24.4 Nein | 24.4 no | Mitarb_nein | 1= yes |
| 24.5 Sonstiges, und zwar: | 24.5 other costumer participate activities: | Mitarb_sonst | open question |
| E: Risikowahrnehmung | E: risk perception |  |  |
| 25. Wie schätzen Sie sich persönlich ein: Sind Sie im Allgemeinen ein risikobereiter Mensch oder versuchen Sie, Risiken zu vermeiden? | 25. What do you think of your risk attitude? Are you rather risk- loving or do you try to minimize risk? | SA | scale from 0 to 10  0= very risk averse  10= very risk loving |
| 26. Durch welche der folgenden Faktoren hatten Sie in den letzten 5 Jahre größere Verluste auf Ihrem Landwirtschaftlichen Betrieb? | 26. Which of the following factors have resulted in a loss on your farm in the past 5 years? |  |  |
| 26.1 Markt- und Preisrisiken | 26.1 market and price risk | Verl_MP | 1= yes |
| 26.2 Risiken durch Politikänderungen | 26.2 risk due to a shift in political framework | Verl_Polit | 1= yes |
| 26.3 Produktionsrisiken | 26.3 production risk | Verl_Prod | 1= yes |
| 26.4 Finanzielle Risiken | 26.4 financial risk | Verl_Fin | 1= yes |
| 26.5 Risiken durch Arbeitskräfte | 26.5 risk regarding workforce | Verl_AK | 1= yes |
| 26.6 Risiken durch gesellschaftliche Akzeptanz | 26.6 risk due to social acceptance | Verl_Akzept | 1= yes |
| 26.7 Keine Verluste aufgrund dieser Faktoren | 26.7 no loss due to these factors | Verl_keine | 1= yes |
| 26.7 Sonstiges, und zwar: | 26.7 other risks: | Verl_sonstige | open question |
| 27. Bitte wählen Sie einen oder mehrere Punkte aus der Liste aus. Wie schätzen Sie die Schadensauswirkungen der folgenden Risikoquellen für Ihren Betrieb ein. | 27. Chose one or several aspects of the given options below. How do you estimate the damage due to the following risk sources for your farm? |  |  |
| 27.1 Markt- und Preisrisiken | 27.1 market and price risk | Riskw_SW_MP | scale from 1 to 5 and in 0=“don’t know”  1= no impact  5= threaten the livelihood |
| 27.2 Risiken durch Politikänderungen | 27.2 risk due to a shift in political framework | Riskw_SW_Polit | scale from 1 to 5 and in 0=“don’t know”  1= no impact  5= threaten the livelihood |
| 27.3 Produktionsrisiken | 27.3 production risk | Riskw_SW_Prod | scale from 1 to 5 and in  0= “don’t know”  1= no impact  5= threaten the livelihood |
| 27.4 Finanzielle Risiken | 27.4 financial risk | Riskw_SW_Fin | scale from 1 to 5 and in  0= “don’t know”  1= no impact  5= threaten the livelihood |
| 27.5 Risiken durch Arbeitskräfte | 27.5 risk regarding workforce | Riskw_SW_AK | scale from 1 to 5 and in  0= “don’t know”  1= no impact  5= threaten the livelihood |
| 27.6 Risiken durch gesellschaftliche Akzeptanz | 27.6 risk due to social acceptance | Riskw_SW_Akzept | scale from 1 to 5 and in  0= “don’t know”  1= no impact  5= threaten the livelihood |
| 28. Wie schätzen Sie die Eintrittswahrscheinlichkeiten der folgenden Risikoquellen für Ihren Betrieb ein? | 28. What is your estimated probability for the following events to occur? |  |  |
| 28.1 Markt und Preisrisiken | 28.1 market and price risk | Riskw_EW_MP | scale from 1 to 5 and in  0= “don’t know”  1= very unlikely  5= highly probably |
| 28.2 Politikänderungen | 28.2 risk due to a shift in political framework | Riskw_EW_Polit | scale from 1 to 5 and in  0= “don’t know”  1= very unlikely  5= highly probably |
| 28.3 Produktionsrisiken | 28.3 production risk | Riskw_EW_Prod | scale from 1 to 5 and in  0= “don’t know”  1= very unlikely  5= highly probably |
| 28.4 Finanzielle Risiken | 28.4 financial risk | Riskw_EW_Fin | scale from 1 to 5 and in  0= “don’t know”  1= very unlikely  5= highly probably |
| 28.5 Risiken durch Arbeitskräfte | 28.5 risk regarding workforce | Riskw_EW_AK | scale from 1 to 5 and in  0= “don’t know”  1= very unlikely  5= highly probably |
| 28.6 Risiken durch gesellschaftliche Akzeptanz | 28.6 risk due to social acceptance | Riskw_EW_Akzept | scale from 1 to 5 and in  0= “don’t know”  1= very unlikely  5= highly probably |
| 29. Bitte geben Sie in der folgenden Tabelle an, inwiefern Sie den Aussagen zustimmen. | 29. How much do you agree with the following statements? |  |  |
| 29.1 Ich bin bereit, in Bezug auf die Produktion mehr Risiken einzugehen als andere Landwirte. | I am willing to take more risks than my colleagues with respect to production. | BS_Prod | scale from 1 to 5:  1= agree (risk- loving)  5= don’t agree (risk- averse) |
| 29.2 Ich bin bereit, in Bezug auf Markt- und Preise mehr Risiken einzugehen als andere Landwirte. | I am willing to take more risks than my colleagues with respect to marketing. | BS_MP | scale from 1 to 5:  1= agree (risk- loving)  5= don’t agree (risk- averse) |
| 29.3 Ich bin bereit, in Bezug auf Fremdkapitalaufnahme mehr Risiken einzugehen als andere Landwirte. | I am willing to take more risks than my colleagues with respect to financial issues. | BS_Fin | scale from 1 to 5:  1= agree (risk- loving)  5= don’t agree (risk- averse) |
| 29.4 Ich bin bereit, in Bezug auf Landwirtschaft generell mehr Risiken einzugehen als andere Landwirte. | I am willing to take more risks than my colleagues with respect to agriculture in general. | BS_gen | scale from 1 to 5:  1= agree (risk- loving)  5= don’t agree (risk- averse) |
| F: Risikopräferenzen | F: Risk preferences |  |  |
| 30. Nehmen Sie an, Ihnen wird angeboten eine landwirtschaftliche Investition zu tätigen. Dabei erhalten Sie mit bestimmten Wahrscheinlichkeiten für Investition A eine Auszahlung von 100.000 € oder 80.000 € und für Investition B eine Auszahlung von 192.500 € oder 5.000 €. Beide Investitionen unterscheiden sich nicht bezüglich der Kosten und Auszahlungszeitpunkte. Sie können in der folgenden Tabelle in jeder Zeile zwischen zwei Investitionsentscheidungen (A oder B) wählen. | 30. Imagine an investment in the agricultural sector is offered to you. You earn with a given probability either 100.000 € or 80.000 € for Investment A and for investment B either 192.500 € or 5.000 €. Both investments have the same costs and payment date. You can chose for each column in the table below between investment A or B. |  |  |
| 30.1 A: 10% für 100.000€ / 90% für 80.000€  B: 10% für 192.500€ / 90% für 5.000€ | 30.1 A: 10% for 100.000€ / 90% for 80.000€  B: 10% for 192.500€ / 90% for 5.000€ | HLL1 | A or B |
| 30.2 A: 20% für 100.000€ / 80% für 80.000€  B: 20% für 192.500€ / 80% für 5.000€ | 30.2 A: 20% for 100.000€ / 80% for 80.000€  B: 20% for 192.500€ / 80% for 5.000€ | HLL2 | A or B |
| 30.3 A: 30% für 100.000€ / 70% für 80.000€  B: 30% für 192.500€ / 70% für 5.000€ | 30.3 A: 30% for 100.000€ / 70% for 80.000€  B: 30% for 192.500€ / 70% for 5.000€ | HLL3 | A or B |
| 30.4 A: 40% für 100.000€ / 40% für 80.000€  B: 40% für 192.500€ / 40% für 5.000€ | 30.4 A: 40% for 100.000€ / 40% for 80.000€  B: 40% for 192.500€ / 40% for 5.000€ | HLL4 | A or B |
| 30.5 A: 50% für 100.000€ / 50% für 80.000€  B: 50% für 192.500€ / 50% für 5.000€ | 30.5 A: 50% for 100.000€ / 50% for 80.000€  B: 50% for 192.500€ / 50% for 5.000€ | HLL5 | A or B |
| 30.6 A: 60% für 100.000€ / 40% für 80.000€  B: 60% für 192.500€ / 60% für 5.000€ | 30.6 A: 60% for 100.000€ / 40% for 80.000€  B: 60% for 192.500€ / 60% for 5.000€ | HLL6 | A or B |
| 30.7 A: 70% für 100.000€ / 30% für 80.000€  B: 70% für 192.500€ / 30% für 5.000€ | 30.7 A: 70% for 100.000€ / 30% for 80.000€  B: 70% for 192.500€ / 30% for 5.000€ | HLL7 | A or B |
| 30.8 A: 80% für 100.000€ / 20% für 80.000€  B: 80% für 192.500€ / 20% für 5.000€ | 30.8 A: 80% for 100.000€ / 20% for 80.000€  B: 80% for 192.500€ / 20% for 5.000€ | HLL8 | A or B |
| 30.9 A: 90% für 100.000€ / 10% für 80.000€  B: 90% für 192.500€ / 10% für 5.000€ | 30.9 A: 90% for 100.000€ / 10% for 80.000€  B: 90% for 192.500€ / 10% for 5.000€ | HLL9 | A or B |
| 30.10 A: 100% für 100.000€ / 0% für 80.000€  B: 100% für 192.500€ / 0% für 5.000€ | 30.10 A: 100% for 100.000€ / 0% for 80.000€  B: 100% for 192.500€ / 0% for 5.000€ | HLL10 | A or B |
| G: Informationen zu Betriebsleitung, Familie und Arbeitskräften | G: Information to farm management, family and worker |  |  |
| 31. Wie viele Personen einschließlich Ihnen leben in Ihrem Haushalt? | 31. How many people, including yourself, live in your household? | HHZ | number of people in household |
| 32. Welche Funktionen nehmen Sie in dem Betrieb ein? | 32. What is your function within the farm? |  |  |
| 32.1 Betriebsleiter/in | 32.1 farm manager | Funktion_farm_manager | 1= yes |
| 32.2 Lebenspartner/in | 32.2 life partner of farm manager | Funktion_life_partner | 1= yes |
| 32.3 Hofnachfolger | 32.3 farm successor | Funktion_farm_management_successor | 1= yes |
| 32.4 Sonstiges | 32.4 other people in household: | Funktion_sonst | open question |
| 33. Wenn Betriebsleiter angekreuzt: In welchem Jahr haben Sie den Betrieb übernommen? | 33. If farm manager, in which year did you take over the farm management? | Erfahrung | year of farm take-over |
| 34. (Nur wenn nicht Hofnachfolger angekreuzt). Ist auf Ihrem Betrieb die Hofnachfolge gesichert? | 34. (Only if not farm successor). Do you have a farm management successor for sure? | HN | 1 = yes, succession secured  0.5 = succession likely  0 = succession not intended in the near future  0.5 = succession unlikely  -1 = no succession secured  -2 =will give up the farm |
| 35. Wie viele Familienarbeitskräfte sind im Betrieb bzw. den Betrieben beschäftigt?  Mit Arbeitsumfang ist die vom jeweiligen Familienmitglied erbrachte Arbeitsleistung (Landwirtschaft+ Diversifizierung) gemeint. Eine Voll- AK entspricht 1,0 AK oder 280 Arbeitstagen pro Jahr. | 35. How many family members are engaged on the farm? Extent of work includes work on farm and diversification of a family member. A full work unit is equal to 1.0 work units or 280 working days. |  |  |
| 35.1 Betriebsleiter Geschlecht | 35.1 sex of farm manager | Betriebsl_sex | 1= male  0= female |
| 35.2 Betriebsleiter Alter | 35.2 age of farm manager | Betriebsl_alter | age of farm manager |
| 35.3 Betriebsleiter Arbeitskraft Landwirtschaft | 35.3 working units of farm manager for farm work | Betriebsl_AK_landw | working units of farm manager for farm work |
| 35.4 Betriebsleiter Arbeitskraft Einkommenskombi | 35.4 working units of farm manager for income diversification | Betriebsl_AK_EKK | working units of farm manager for income diversification |
| 35.5 Lebenspartner Geschlecht | 35.5 sex of life partner | Partner_sex | 1= male  0= female |
| 35.6 Lebenspartner Alter | 35.6 age of life partner | Partner_alter | age of life partner |
| 35.7 Lebenspartner Arbeitskraft Landwirtschaft | 35.7 working units of life partner for farm work | Partner_AK_landw | working units of life partner for farm work |
| 35.8 Lebenspartner Arbeitskraft Einkommenskombi | 35.8 working units life partner for income diversification | Partner_AK_EKK | working units of life partner for income diversification |
| 35.9 Hofnachfolger Geschlecht | 35.9 sex of farm successor | HN_sex | 1= male  0= female |
| 35.10 Hofnachfolger Alter | 35.10 age of farm management successor | HN_alter | age of farm management successor |
| 35.11 Hofnachfolger Arbeitskraft Landwirtschaft | 35.11 working units of farm management successor for farm work | HN_AK_landw | working units of farm successor for farm work |
| 35.12 Hofnachfolger Arbeitskraft Einkommenskombi | 35.12 working units of farm management successor for income diversification | HN_AK_EKK | working units off arm management successor for income diversification |
| 35.13 Kind1 Geschlecht | 35.13 sex of child1 | Kind1_sex | 1= male  0= female |
| 35.14 Kind1 Alter | 35.14 age of child1 | Kind1_alter | age of child1 |
| 35.15 Kind1 Arbeitskraft_ Landwirtschaft | 35.15 working units of child1 for farm work | Kind1_AK_landw | working units of child1 for farm work |
| 35.16 Kind1 Arbeitskraft Einkommenskombi | 35.16 working units child1 for income diversification | Kind1_AK_EKK | working units of child1 for income diversification |
| 35.17 Kind2 Geschlecht | 35.17 sex of child2 | Kind2_sex | 1= male  0= female |
| 35.18 Kind2 Alter | 35.18 age of child2 | Kind2_alter | age of child2 |
| 35.19 Kind2 Arbeitskraft Landwirtschaft | 35.19 working units of child2 for farm work | Kind2_AK_landw | working units of child2 for farm work |
| 35.20 Kind2 Arbeitskraft Einkommenskombi | 35.20 working units child2 for income diversification | Kind2_AK_EKK | working units of child2 for income diversification |
| 35.21 Kind3 Geschlecht | 35.21 sex of child3 | Kind3_sex | 1= male  0= female |
| 35.22 Kind3 Alter | 35.22 age of child3 | Kind3_alter | age of child3 |
| 35.23 Kind3 Arbeitskraft Landwirtschaft | 35.23 working units of child3 for farm work | Kind3_AK_landw | working units of child3 for farm work |
| 35.24 Kind3 Arbeitskraft Einkommenskombi | 35.24 working units child3 for income diversification | Kind3_AK_EKK | working units of child3 for income diversification |
| 36. Sind familienfremde Arbeitskräfte auf dem Betrieb/ den Betrieben beschäftigt? | 36. Are there any non-family members employed on the farm? | FAK | 1= yes  0= no |
| 37. Wenn 36 ja, wie viele Fremdarbeitskräfte sind in ihrem Betrieb durchschnittlich beschäftigt? Anzahl der Fremdarbeitskräfte | 37. If 36 with yes, how many non- family employees do you have on average on your farm? Number of non-family employees | FAK_anzahl | number of non- family employees |
| 38. Wenn 36 ja, Mit welchem Arbeitsumfang sind die Fremdarbeitskräfte auf Ihrem Betrieb bzw. den Betrieben beschäftigt? | 38. If 36 with yes, how many working units do the non-family members have? |  |  |
| 38.1 Arbeitsumfang Landwirtschaft (AK) | 38.1 working unit agriculture | FAK_AK_landw | working units of non- family employees for farm work |
| 38.2 Arbeitsumfang Diversifizierung (AK) | 38.2 working unit diversification | FAK_AK_EKK | working units if non- family employees for income diversification |
| 39.1 Bitte geben Sie Ihren höchsten Bildungsabschluss an: | 39.1 What is your highest qualification? | edu | highest educational degree in German schooling system  1= no degree^[[1]](#footnote-1)^  2= secondary school certificate (9 years)^[[2]](#footnote-2)^  3= secondary school certificate (11 years)^[[3]](#footnote-3)^  4= advanced technical college certificate^[[4]](#footnote-4)^  5= high school diploma^[[5]](#footnote-5)^  6= completed vocational training^[[6]](#footnote-6)^  7= certified manager^[[7]](#footnote-7)^  8= certified agriculturist^[[8]](#footnote-8)^  9= masters certificate in agriculture^[[9]](#footnote-9)^ |
| 39.2 Sonstiges: | 39.2 other degree: | edu_sonst | open question |
| 40. Wie hat sich Ihr Haushaltseinkommen im Durchschnitt der letzten drei Jahre zusammengesetzt? | 40. How has your household income been compounded on average in the past three years? |  |  |
| 40.1 Einkommen aus der eigentlichen landwirtschaftlichen Tätigkeit? | 40.1 income derived from agricultural activities | HHEK_landw | share of income from agricultural activities in% of total income |
| 40.2 Einkommen aus Diversifizierung | 40.2 income derived from diversification | HHEK_EKK | share of income from diversification in% of total income |
| 40.3 Einkommen aus nicht- selbstständiger Tätigkeit | 40.3 income derived from non- self-employment | HHEK_nicht_selbst_Taet | share of income from non- self- employment in% of total income |
| 40.4 Einkommen aus sonstigen Einkünften | 40.4 income derived from other sources | HHEK_sonst | share of income from other sources in% of total income |
| H: Zukunftsprognosen | H: Forecast for the future |  |  |
| 41. Wie schätzen Sie selbst Ihre derzeitige betriebliche Situation ein? | 41. How do you perceive the current situation of the farm? | Betriebssituation | scale from 0 to 100  0= very negative  100= very positive |
| 42. Können Sie für Ihren Betrieb ein Alleinstellungsmerkmal benennen, welches Sie von anderen Betrieben unterscheidet? | 42. Do you have a unique selling proposition in comparison to other farms? | USP | 1= yes |
| 43 Wenn ja: Bitte benennen Sie das Alleinstellungsmerkmal Ihres Betriebes? | 43. If yes, please name your unique selling proposition: | USP_welcher | open question |
| 44. Wie sieht Ihre zukünftige Betriebsstrategie (Landwirtschaft + Diversifizierung) aus? Bitte geben Sie jeweils Ihre Strategie für beide Bereiche (Landwirtschaft+ Diversifizierung) an. | 44. What is your future corporate strategy? Please name the future strategy for both agriculture and diversification. |  |  |
| 44.1 Landwirtschaft | 44.1 agricultural activities | Zuk_landw | 1= invest/ grow  2= consolidate  3= new business branch  4= reduce  5= close business branch  6= quit completely |
| 44.2 Diversifikation | 44.2 diversification | Zuk_EKK | 1= invest/ grow  2= consolidate  3= new business branch  4= reduce  5= close business branch  6= quit completely |
| 45. Haben Sie Interesse an diesem Ergebnisbericht? | 45. Are you interested in the results of this survey? | Bericht1 | 1= yes, interested in survey |
| 46. Möchten Sie an der Verlosung des Gewinnspiels als Dankeschön für Ihre Teilnahme mitmachen? | 46. Do you want to participate in the price draw? | Bericht2 | 1= yes, participate in price draw |
| 47. Wenn 45 ja: Bitte teilen Sie uns Ihre E-Mail Adresse | 47. If question 45 has been answered with yes, please tell us your e-mail address. | Bericht3 | e-mail address |
| 48. Wie lautet Ihre Adresse? | 48. What is your address? | Bericht4 | address of farm |
|  |  |  |  |
|  |  | interviewtime | total time to complete online survey in seconds |
|  |  | timeBetrieb | time to complete first part of survey in seconds |
|  |  | timeLage | time to complete second part of survey in seconds |
|  |  | timeBer | time to complete third part of survey in seconds |
|  |  | timeEKK | time to complete fourth part of survey in seconds |
|  |  | timeRisk | time to complete fifth part of survey in seconds |
|  |  | timeHLL | time to complete sixth part of survey in seconds |
|  |  | timePers | time to complete seventh part of survey in seconds |
|  |  | timeZukunft | time to complete eight part of survey in seconds |
|  |  | timeBericht | time to complete ninth part of survey in seconds |
|  |  | Bodenfruchtbarkeit | 0-100  0=very poor soil fertility (min)  100=very good soil fertility (max) |
|  |  | Distanz zu erster/zweiter Ordnung aggregiert | Distance in meters between farm and main roads (no highways „Autobahnen“) |
|  |  | Entfernung.zu.Siedlung.Rand.(m) | Distance in meters between farm and next city of > 100,000 inhabitants (distance to the settlement border outwards) |
|  |  | Distanz.zu.Straßen.erster.Ordnung.(m) | Distance in meters between farm and main roads first order (no highways „Autobahnen“, but next lower category; federal roads “Bundesstraßen”) |
|  |  | Distanz.zu.Straßen.zweiter.Ordnung.(m) | Distance in meters between farm and main roads first order (no highways „Autobahnen“, but 2^nd^ next lower category; country roads “Landstraßen”) |

1. Schule beendet ohne Abschluss [↑](#footnote-ref-1)
2. Hauptschulabschluss [↑](#footnote-ref-2)
3. Realschulabschluss [↑](#footnote-ref-3)
4. Fachhochschulreife [↑](#footnote-ref-4)
5. Allgemeine Hochschulreife [↑](#footnote-ref-5)
6. Berufsausbildung/Lehre [↑](#footnote-ref-6)
7. Staatlich geprüfter Wirtschafter [↑](#footnote-ref-7)
8. Staatlich geprüfter Agrarbetriebswirt (Landwirt) [↑](#footnote-ref-8)
9. Landwirtschaftsmeister [↑](#footnote-ref-9)
