# Supplementary material for: Data on farm diversification decisions and farmers’ risk preferences in the Ruhr Metropolitan region (Germany)
Source: Data Brief. 2018 Mar 7;18:9–12. doi: 10.1016/j.dib.2018.03.008 (PMC5996160; doi:10.1016/j.dib.2018.03.008)
Supplement: Supplementary file 4 — Supplementary material Appendix 3: The survey (in German). [file mmc4.docx]

**Appendix 3: The survey (in German)**

**Befragung zur urbanen Landwirtschaft im Ruhrgebiet**

**Frageblock 1: Betriebliche Informationen**

1. Betreiben Sie Ihren Betrieb im Haupt- oder Nebenerwerb?

☐ Haupterwerb (mind. 50 % des Einkommens aus der Landwirtschaft)

☐ Nebenerwerb

1. Wirtschaften Sie konventionell oder nach ökologischen Richtlinien?

☐ Zertifiziert ökologischer Landbau

☐ In Umstellung

☐ Koventionell

1. Wie ist der aktuelle Stand Ihrer Flächenausstattung?

Landwirtschaftsfläche insgesamt ___ha

Ackerland ___ha

Grünland ___ha

1. Wie viel Hektar haben Sie derzeit gepachtet? ___ha
2. Welche Kulturen bauen Sie derzeit in welchem Umfang an?

☐ Getreide __ha

☐ Hackfrüchte __ha

☐ Sonderkulturen __ha

☐ Dauerkulturen __ha

☐ Futterpflanzen __ha

☐ Hülsenfrüchte __ha

☐ Ölsaaten __ha

☐ Grünland __ha

☐ Sonstiges: ___________________ __ha

1. Bauen Sie Kulturen in Gewächshäusern und/oder unter Folien an?

☐ Nein

☐ Ja, Gewächshäuser: Wie viel m² kultivieren Sie unter Glas? ___m²

☐ Ja, Folien: Wie viel m² kultivieren Sie unter Folien? ___m²

1. Welche Tiere halten Sie derzeit in Ihnen im Betrieb mit welchem Bestand?

☐ Mutterkühe __Tiere

☐ Fleischrinder/Bullen __Tiere

☐ Sauen __Tiere

☐ Schweinemast __Tiere

☐ Legehennen __Tiere

☐ Geflügelmast __Tiere

☐ Schafe/Ziegen __Tiere

☐ Pferde __Tiere

☐ Sonstiges: ___________________ __Tiere

**Frageblock 2: Lagebezug**

1. Wie stark unterscheiden sich Ihrer Meinung nach die städtische und ländliche Landwirtschaft?

Skala von 0 – 100 (10 cm) als Regler (bei Online-Befragung)

[0 = keine Unterschiede, 100 = sehr große Unterschiede]

1. Liegt Ihr Betrieb in einer eher städtischen oder ländlichen Umgebung?

Skala von 0 – 100 (10 cm) als Regler [0 = sehr ländlich, 100 = sehr städtisch]

1. Bietet das städtische Umfeld für Ihre Betriebsausrichtung mehrheitlich Vor- oder Nachteile?

Skala von 0 – 100 (10 cm) als Regler [0 = nur Vorteile, 100 = nur Nachteile]

1. Welche Vorteile eines städtischen Umfelds fallen Ihnen für Ihre Betriebsausrichtung ein? (Mehrfachauswahl möglich)

☐ Großer Verbrauchermarkt in der Region

☐ Kaufkraft in der Region

☐ Gute Infrastruktur (Verkehr, Medien)

☐ Vor- und nachgelagerte Industrie in der Region

☐ Urbane Ressourcen nutzbar (Wärme, organische Abfälle etc.)

☐ Sonstiges, und zwar:___________________

1. Welche Nachteile eines städtischen Umfelds fallen Ihnen für Ihre Betriebsausrichtung ein? (Mehrfachauswahl möglich)

☐ Land: Flächenknappheit und weiterer Flächenverlust

☐ Land: Flächenzersplitterung

☐ Hoher Pachtflächenanteil mit kurzen Laufzeiten

☐ Akzeptanzprobleme, kritische Nachbarschaft

☐ Vandalismus, Diebstahl

☐ Sonstiges, und zwar:___________________

1. Bitte geben Sie Ihre Postleitzahl an:

|  |  |  |  |  |
| --- | --- | --- | --- | --- |

**Frageblock 3: Beratung**

1. Nutzen Sie derzeit oder haben Sie in den letzten 5 Jahren Beratungsangebote in Anspruch genommen?

☐ Ja

☐ Nein

1. (Wenn Frage 14 ja )Welche Beratungsangebote der LWK haben Sie in den letzten 5 Jahren in Anspruch genommen?

☐ Beratung zur Unternehmensführung

☐ Beratung zu Fördermaßnahmen

☐ Beratung im Ackerbau

☐ Beratung zur Rinderhaltung

☐ Beratung zur Schweinehaltung

☐ Beratung zur Geflügelhaltung

☐ Beratung zur Pferdehaltung

☐ Beratung zur Schafhaltung

☐ Beratung im Ökologischen Landbau

☐ Landservice-Beratung (Diversifizierungsangebote)

☐ Beratung im Gartenbau

☐ Beratung zur Wasserrahmenrichtlinie

☐ Beratung zum Klimaschutz

1. Wäre Ihrer Meinung nach eine Beratung speziell für Landwirtschaftsbetriebe im städtischen Umfeld geeignet oder nicht?

Skala von 0 – 100 (10 cm) als Regler [0 = ungeeignet, 100 = sehr geeignet]

1. Wäre Ihrer Meinung nach eine Beratungsregion „Metropole Ruhr“ geeignet oder nicht?

Skala von 0 – 100 (10 cm) als Regler [0=sehr ungeeignet, 100=sehr geeignet]

**Frageblock 4: Vermarktung und Diversifizierung**

1. Welche Art von Einkommenskombinationen nutzen Sie in Ihrem Betrieb?

**Tourismus**

☐ Gastronomie

☐ Urlaub (Beherbergung)

☐ Vermietung von Räumlichkeiten für Ferien und Tagungen

☐ Pferdehof/Pensionspferde

☐ Veranstaltungen, Sport- oder Freizeitaktivitäten (Spielangebote für Kinder,

Führungen, Fahrrad- und Kanuverleih etc.)

☐ Sonstiges, und zwar:_______

Bitte geben Sie für die angekreuzten Aktivitäten an, seit welchem Jahr Sie diese betreiben.

Angekreuzte Aktivität Seit dem Jahre __________

**Dienstleistung**

☐ Soziale Dienstleitungen

☐ Land-/Forst-/Hauswirtschaftliche Dienstleistungen (kommunale und private)

☐ Sonstiges, und zwar:_______

Bitte geben Sie für die angekreuzten Aktivitäten an, seit welchem Jahr Sie diese betreiben.

Angekreuzte Aktivität Seit dem Jahre __________

**Erneuerbare Energien**

☐ Nachwachsende Rohstoffe (Biomasse)

☐ Nicht-nachwachsende Rohstoffe (Sonne, Wind, Erdwärme, etc.)

Bitte geben Sie für die angekreuzten Aktivitäten an, seit welchem Jahr Sie diese betreiben.

Angekreuzte Aktivität Seit dem Jahre __________

**Weiterverarbeitung landwirtschaftlicher Produkte**

☐ Weiterverarbeitung pflanzlicher Produkte

☐ Weiterverarbeitung tierischer Produkte

Verkaufen Sie die weiterverarbeiteten pflanzlichen/tierischen Produkte direkt?

☐ Ja, komplett

☐ Ja, teilweise

☐ Nein

**Direktvermarktung (Hofladen, Verkaufswagen)**

☐ Hofladenverkauf

☐ Verkaufswagen (Lieferdienst)

☐ Verkaufsstand (Bauern-/Wochenmarkt)

☐ Verkaufsstand (Straße)

☐ Verkaufsautomat

☐ Liefer-/Partyservice

☐ Sonstiges, und zwar:_______

Bitte geben Sie für die angekreuzten Aktivitäten an, seit welchem Jahr Sie diese betreiben.

Angekreuzte Aktivität Seit dem Jahre _________

1. Mit ca. welchen Anteilen vermarkten Sie Ihre Produkte?

Direkte Vermarktung mit ca. ___%

Indirekte Vermarktung mit ca. ___%

1. Welche Kanäle verwenden Sie für Ihre indirekte Produktvermarktung?

☐ Großmarkt

☐ Genossenschaften

☐ Regionaler Einzelhandel (Supermarkt, Discounter)

☐ Weiterverarbeitender Zwischenhändler (Metzger, Bäcker, Gastronomie etc.)

☐ Erzeugergemeinschaft

☐ Sonstiges, und zwar:_______

1. Sonstige Einkommenskombinationen: ___________________
2. Keine Einkommenskombination ☐
3. Können bei Ihnen Kunden aktiv in der Produktion bzw. im Management aktiv partizipieren/teilhaben?

☐ Ja, Selbstpflückfelder

☐ Ja, Mietgärten

☐ Ja, Solidarische Landwirtschaft

☐ Nein

☐ Sonstiges, und zwar: ___________________

**Frageblock 5: Risikowahrnehmung**

1. Wie schätzen Sie sich persönlich ein: Sind Sie im Allgemeinen ein risikobereiter Mensch oder versuchen Sie, Risiken zu vermeiden?

|  | Gar nicht risiko-bereit | |  | | |  | |  | |  | |  | |  | | Sehr ririsiko-bbereit | |
| --- | --- | --- | --- | --- | --- | --- | --- | --- | --- | --- | --- | --- | --- | --- | --- | --- | --- |
|  | **1** | **2** | | **3** | **4** | | **5** | **6** | **7** | | **8** | | **9** | | **10** | |  |
| Risiko-einschätzung | ☐ | ☐ | | ☐ | ☐ | | ☐ | ☐ | ☐ | | ☐ | | ☐ | | ☐ | |  |

1. Durch welche der folgenden Faktoren hatten Sie in den letzten 5 Jahren größere Verluste auf Ihrem landwirtschaftlichen Betrieb (Mehrfachnennung möglich)?

☐ Markt- und Preisrisiken

☐ Politikänderungen

☐ Produktionsrisiken

☐ Finanzielle Risiken

☐ Risiken durch Arbeitskräfte

☐ Risiken durch gesellschaftliche Akzeptanz

☐ keine größeren Verluste in den letzten 5 Jahren

☐ Sonstiges, und zwar: _____________________

1. Bitte wählen Sie einen oder mehrere Punkte aus der Liste aus. Wie schätzen Sie die Schadensauswirkungen der folgenden Risikoquellen für Ihren Betrieb ein (von 1 = keine Auswirkungen, 5 = existenzgefährdend)?

|  | Keine Auswirkungen | |  | | | Existenz-gefährdend | | Weiß nicht | |
| --- | --- | --- | --- | --- | --- | --- | --- | --- | --- |
|  | **1** | **2** | | **3** | **4** | | **5** |  |  |
| Markt- und Preisrisiken | ☐ | ☐ | | ☐ | ☐ | | ☐ | ☐ |  |
| Risiken durch Politikänderungen | ☐ | ☐ | | ☐ | ☐ | | ☐ | ☐ |  |
| Produktionsrisiken | ☐ | ☐ | | ☐ | ☐ | | ☐ | ☐ |  |
| Finanzielle Risiken | ☐ | ☐ | | ☐ | ☐ | | ☐ | ☐ |  |
| Risiken durch Arbeitskräfte, | ☐ | ☐ | | ☐ | ☐ | | ☐ | ☐ |  |
| Risiken durch gesellschaftliche Akzeptanz | ☐ | ☐ | | ☐ | ☐ | | ☐ | ☐ |  |

1. Wie schätzen Sie die Eintrittswahrscheinlichkeit der folgenden Risikoquellen für Ihren Betrieb ein(von 1 = „sehr unwahrscheinlich“, 5 = „sehr wahrscheinlich“)?

|  | | Keine Auswirkungen | | | |  | | | | existenzgefährdend | | | | Weiß nicht | |
| --- | --- | --- | --- | --- | --- | --- | --- | --- | --- | --- | --- | --- | --- | --- | --- |
|  | | **1** | **2** | | **3** | | | **4** | | | | **5** | |  | |
| Markt- und Preisrisiken | | ☐ | ☐ | | ☐ | | | ☐ | | | | ☐ | | ☐ | |
| Politikänderungen | | ☐ | ☐ | | ☐ | | | ☐ | | | | ☐ | | ☐ | |
| Produktionsrisiken | | ☐ | ☐ | | ☐ | | | ☐ | | | | ☐ | | ☐ | |
| Finanzielle Risiken | | ☐ | ☐ | | ☐ | | | ☐ | | | | ☐ | | ☐ | |
| Risiken durch Arbeitskräfte, | | ☐ | ☐ | | ☐ | | | ☐ | | | | ☐ | | ☐ | |
| Risiken durch gesellschaftliche Akzeptanz | ☐ | | | ☐ | | | ☐ | | ☐ | | ☐ | | ☐ | |  |

1. Bitte geben Sie in der folgenden Tabelle an inwiefern Sie den Aussagen zustimmen (von 1 = „stimme voll zu“ bis 5 = „lehne ab“).

|  | stimme voll zu |  |  |  | lehne ab |
| --- | --- | --- | --- | --- | --- |
|  | **1** | **2** | **3** | **4** | **5** |
| Ich bin bereit, in Bezug auf die Produktion mehr Risiken einzugehen als andere Landwirte. | ☐ | ☐ | ☐ | ☐ | ☐ |
| Ich bin bereit, in Bezug auf Markt- und Preise mehr Risiken einzugehen als andere Landwirte. | ☐ | ☐ | ☐ | ☐ | ☐ |
| Ich bin bereit, in Bezug auf Fremdkapitalaufnahme mehr Risiken einzugehen als andere Landwirte. | ☐ | ☐ | ☐ | ☐ | ☐ |
| Ich bin bereit, in Bezug auf Landwirtschaft generell mehr Risiken einzugehen als andere Landwirte. | ☐ | ☐ | ☐ | ☐ | ☐ |

**Frageblock 6: Risikopräferenzen**

Diese Frage wird zur Auslosung Ihres Gewinns genutzt. Es werden am Ende 10% der Teilnehmer als Gewinner ausgelost. Nähere Details darüber wie Ihr Gewinn ermittelt wird finden Sie [hier](http://ilr.uni-bonn.de/pe/pdf/Umfrage/gewinn%20ruhrgebiet.pdf)

Bitte entscheiden Sie sich in jeder Zeile für eine Investition in A oder B.

1. Nehmen Sie an, Ihnen wird angeboten eine landwirtschaftliche Investition zu tätigen. Dabei erhalten Sie mit bestimmten Wahrscheinlichkeiten für Investition A eine Auszahlung von 100.000 € oder 80.000 € und für Investition B eine Auszahlung von 192.500 € oder 5.000 €. Beide Investitionen unterscheiden sich nicht bezüglich der Kosten und Auszahlungszeitpunkte. Sie können in der folgenden Tabelle in jeder Zeile zwischen den zwei Investitionsentscheidungen (A oder B) wählen.

|  | A | B | A | B |
| --- | --- | --- | --- | --- |
| 1 | 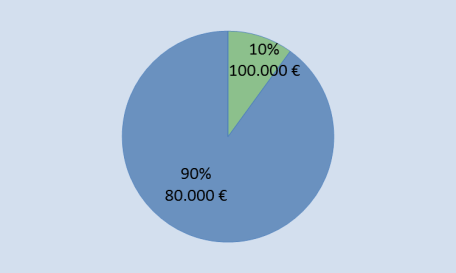10% Wahrscheinlichkeit für eine Auszahlung von 100.000 € und 90% Wahrscheinlichkeit für eine Auszahlung von 80.000 € | 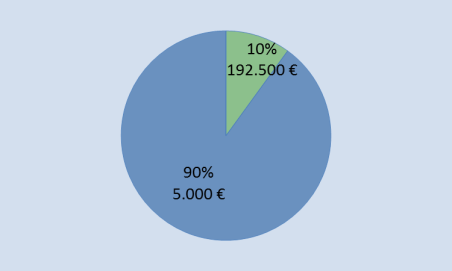10% Wahrscheinlichkeit für eine Auszahlung von 192.500 € und 90% Wahrscheinlichkeit für eine Auszahlung von 5.000 € | ☐ | ☐ |
| 2 | 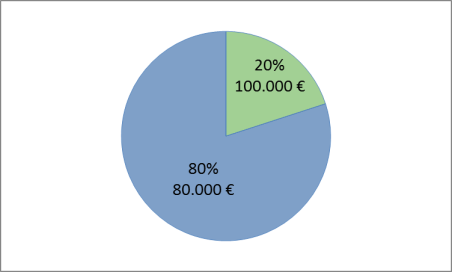20% Wahrscheinlichkeit für eine Auszahlung von 100.000 € und 80% Wahrscheinlichkeit für eine Auszahlung von 80.000 € | 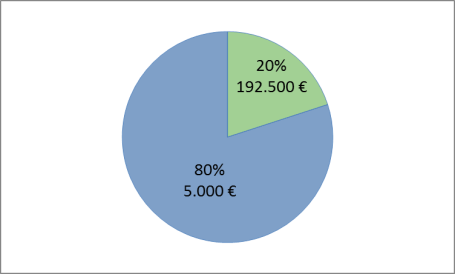20% Wahrscheinlichkeit für eine Auszahlung von 192.500 € und 80% Wahrscheinlichkeit für eine Auszahlung von 5.000 € | ☐ | ☐ |
| 3 | 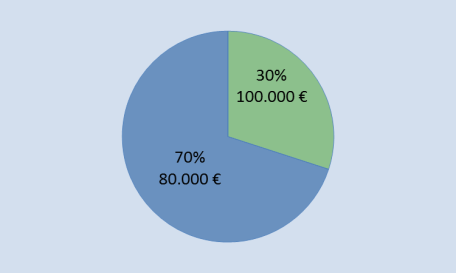30% Wahrscheinlichkeit für eine Auszahlung von 100.000 € und 70% Wahrscheinlichkeit für eine Auszahlung von 80.000 € | 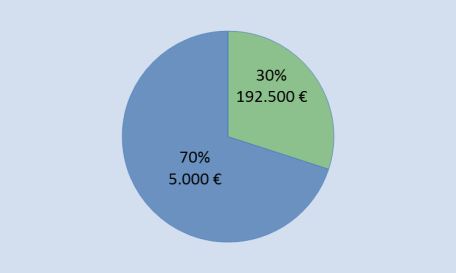30% Wahrscheinlichkeit für eine Auszahlung von 192.500 € und 70% Wahrscheinlichkeit für eine Auszahlung von 5.000 € | ☐ | ☐ |
| 4 | 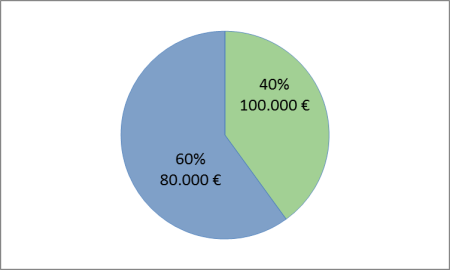40% Wahrscheinlichkeit für eine Auszahlung von 100.000 € und 60% Wahrscheinlichkeit für eine Auszahlung von 80.000 € | 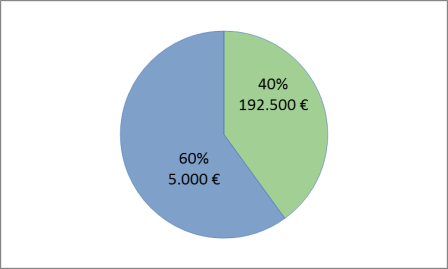40% Wahrscheinlichkeit für eine Auszahlung von 192.500 € und 60% Wahrscheinlichkeit für eine Auszahlung von 5.000 € | ☐ | ☐ |
| 5 | 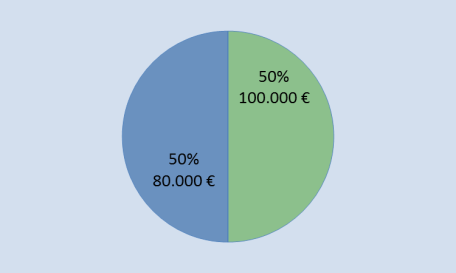50% Wahrscheinlichkeit für eine Auszahlung von 100.000 € und 50% Wahrscheinlichkeit für eine Auszahlung von 80.000 € | 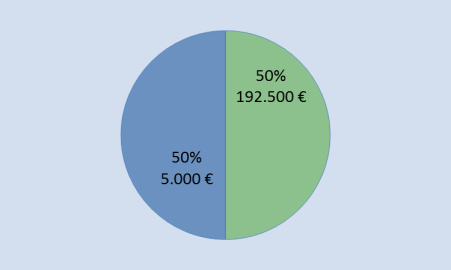50% Wahrscheinlichkeit für eine Auszahlung von 192.500 € und 50% Wahrscheinlichkeit für eine Auszahlung von 5.000 € | ☐ | ☐ |
| 6 | 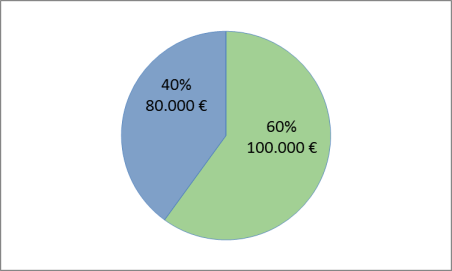60% Wahrscheinlichkeit für eine Auszahlung von 100.000 € und 40% Wahrscheinlichkeit für eine Auszahlung von 80.000 € | 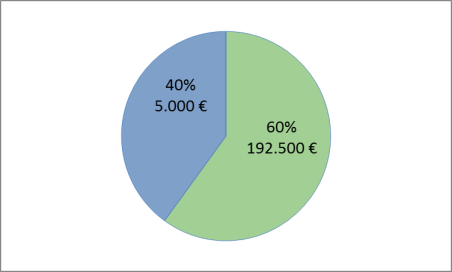60% Wahrscheinlichkeit für eine Auszahlung von 192.500 € und 40% Wahrscheinlichkeit für eine Auszahlung von 5.000 € | ☐ | ☐ |
| 7 | 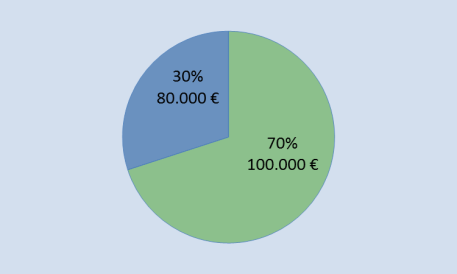70% Wahrscheinlichkeit für eine Auszahlung von 100.000 € und 30% Wahrscheinlichkeit für eine Auszahlung von 80.000 € | 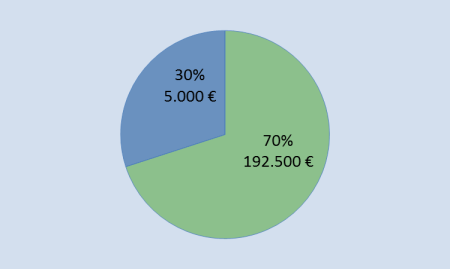70% Wahrscheinlichkeit für eine Auszahlung von 192.500 € und 30% Wahrscheinlichkeit für eine Auszahlung von 5.000 € | ☐ | ☐ |
| 8 | 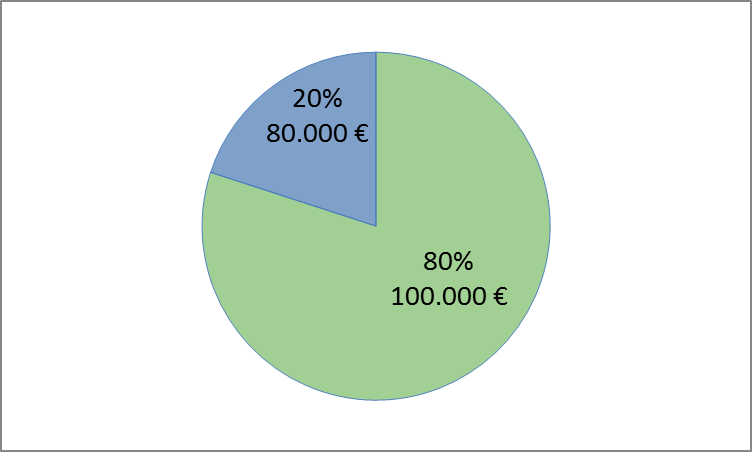80% Wahrscheinlichkeit für eine Auszahlung von 100.000 € und 20% Wahrscheinlichkeit für eine Auszahlung von 80.000 € | 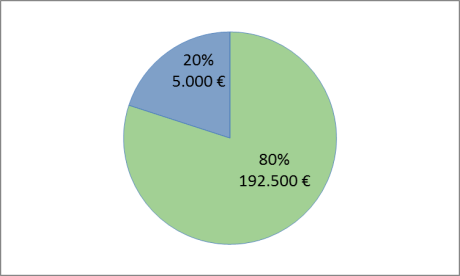80% Wahrscheinlichkeit für eine Auszahlung von 192.500 € und 20% Wahrscheinlichkeit für eine Auszahlung von 5.000 € | ☐ | ☐ |
| 9 | 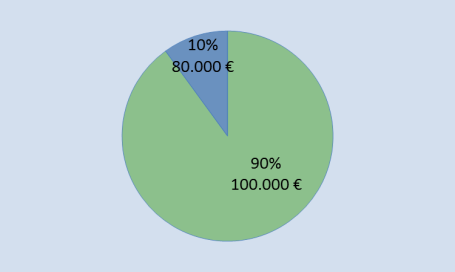90% Wahrscheinlichkeit für eine Auszahlung von 100.000 € und 10% Wahrscheinlichkeit für eine Auszahlung von 80.000 € | 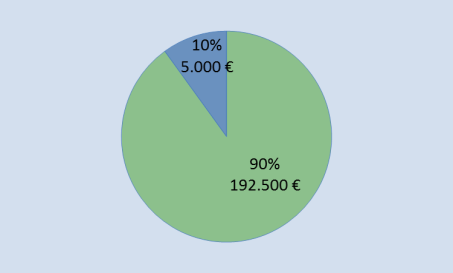90% Wahrscheinlichkeit für eine Auszahlung von 192.500 € und 10% Wahrscheinlichkeit für eine Auszahlung von 5.000 € | ☐ | ☐ |
| 10 | 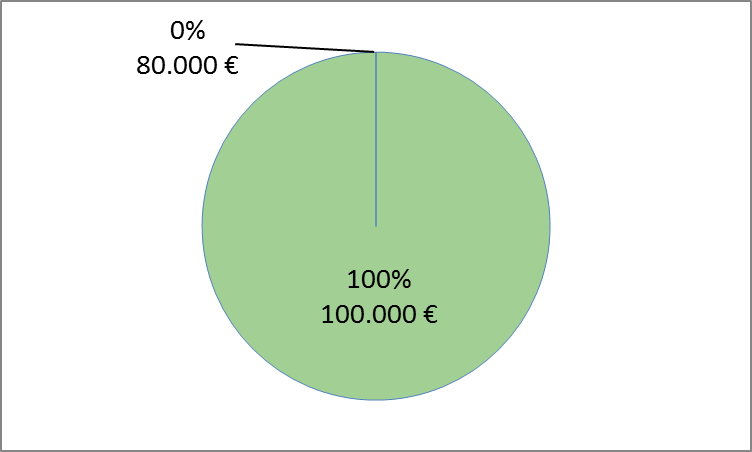100% Wahrscheinlichkeit für eine Auszahlung von 100.000 € und 0% Wahrscheinlichkeit für eine Auszahlung von 80.000 € | 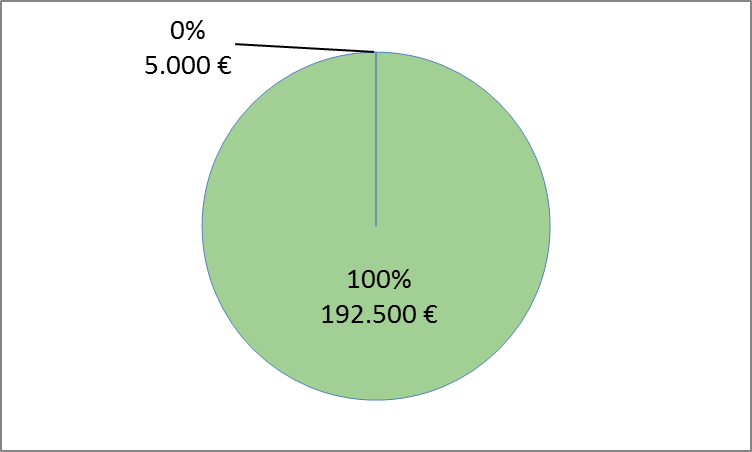100% Wahrscheinlichkeit für eine Auszahlung von 192.500 € und 0% Wahrscheinlichkeit für eine Auszahlung von 5.000 € | ☐ | ☐ |

Bitte entscheiden Sie sich **in jeder Zeile** für eine Investition in A oder B.

**Frageblock 7: Informationen zu Betriebsleitung, Familie und Arbeitskräften**

1. Wie viele Personen einschließlich Ihnen leben in Ihrem Haushalt?

 Personen

1. Welche Funktion nehmen Sie in dem Betrieb ein?

☐ Betriebsleiter/in

☐ Lebenspartner/in

☐ Hofnachfolger

☐ Sonstiges

1. Wenn Betriebsleiter angekreuzt:

In welchem Jahr haben Sie den Betrieb übernommen? _______________

1. (Nur wenn nicht Hofnachfolger angekreuzt)

Ist auf Ihrem Betrieb die Hofnachfolge gesichert?

| ☐ja, gesichert | ☐ja, wahrscheinlich gesichert | ☐nein, eher nicht gesichert | ☐ nein, völlig unsicher | | | | |
| --- | --- | --- | --- | --- | --- | --- | --- |
|  | | | |  |  |  |  |
| ☐ nein, es steht keine Hofübernahme in überschaubarer Zeit an | | | | | | |  |
| ☐ nein, auslaufender Betrieb | | | | | | |  |

1. Wie viele Familienarbeitskräfte sind im Betrieb bzw. den Betrieben beschäftigt?

Mit Arbeitsumfang ist die vom jeweiligen Familienmitglied erbrachte Arbeitsleistung (Landwirtschaft + Diversifizierung) gemeint. Eine Voll-AK entspricht 1,0 AK oder 280 Arbeitstage pro Jahr.

| Personen | Geschlecht  m/w | Alter | Arbeitsumfang  (Angabe in AK) | |
| --- | --- | --- | --- | --- |
|  |  |  | Landwirtschaft | Diversifizierung |
| Betriebsleiter/in |  |  |  |  |
| Lebenspartner/in |  |  |  |  |
| Kind 1 |  |  |  |  |
| Kind 2 |  |  |  |  |
| Kind 3 |  |  |  |  |

1. Sind familienfremde Arbeitskräfte auf dem Betrieb/ den Betrieben beschäftigt?

☐ Ja

☐ Nein

1. Wenn 35 Ja: **Wie viele Fremdarbeitskräfte sind in Ihrem Betrieb durchschnittlich beschäftigt?**

Anzahl der Fremdarbeitskräfte: ___________________

1. **Wenn 35 ja: Mit welchem Arbeitsumfang sind die Fremdarbeitskräfte auf Ihrem Betrieb bzw. den Betrieben beschäftigt?**

|  | Arbeitsumfang  Landwirtschaft (AK) | Arbeitsumfang  Diversifizierung (AK) |
| --- | --- | --- |
| Summe Fremdarbeitskräfte |  |  |

1. Bitte geben Sie Ihren höchsten Bildungsabschluss an:

| ☐ | Schule beendet ohne Abschluss |
| --- | --- |
| ☐ | Hauptschulabschluss |
| ☐ | Realschulabschluss |
| ☐ | Fachhochschulreife |
| ☐ | Allgemeine Hochschulreife |

| ☐ | Berufsausbildung/Lehre |
| --- | --- |
| ☐ | Staatlich geprüfter Wirtschafter |
| ☐ | Staatlich geprüfter Agrarbetriebswirt (Landwirt) |
| ☐ | Landwirtschaftsmeister |
| ☐ | Hochschulabschluss (Unioder FH) |
| ☐ | Anderer Schulabschluss ………………………… |

1. Wie hat sich Ihr Haushaltseinkommen im Durchschnitt der letzten drei Jahrezusammengesetzt?

Bitte tragen Sie wenn nicht zutreffend 0% ein.

Hier interessieren wir uns für Ihre Einschätzung, welchen Anteil Ihre einzelnen Aktivitäten zu Ihrem

gesamten Einkommen beitragen. Bitte beachten Sie, dass in der Summe 100 % erreicht werden.

| Einkommen aus | Anteil |
| --- | --- |
| … der eigentlichen landwirtschaftlichen Tätigkeit | % |
| …Diversifizierung | % |
| …nicht-selbständiger Tätigkeit | % |
| …sonstigen Einkünften | % |
| Verbleibend | 100 % |
| Gesamt | 0% |

**Frageblock 8: Zukunftsprognosen**

1. Wie schätzen Sie selbst Ihre derzeitige betriebliche Situation ein?

Skala von 0 – 100 (10 cm) als Regler [0 = sehr negativ, 100 = sehr positiv]

1. Können Sie für Ihren Betrieb ein Alleinstellungsmerkmal benennen, welches Sie von anderen Betrieben unterscheidet?

☐ Nein

☐ Ja

1. Wenn ja: Bitte benennen Sie das Alleinstellungsmerkmal Ihres Betriebes?___________________
2. Wie sieht Ihre zukünftige Betriebsstrategie (Landwirtschaft+ Diversifizierung) aus?

Bitte geben Sie jeweils Ihre Strategie für beide Bereiche (Landwirtschaft + Diversifizierung) an!

|  | Wachsen/  Investieren | Konsoli-dieren | Neue Zweige aufbauen | Reduzieren | Bereiche einstellen | Ganz aussteigen |
| --- | --- | --- | --- | --- | --- | --- |
| Landwirt-schaft | ☐ | ☐ | ☐ | ☐ | ☐ | ☐ |
| Diversi-fizierung | ☐ | ☐ | ☐ | ☐ | ☐ | ☐ |

1. Haben Sie abschließend noch Anmerkungen?

________________________________________

1. Haben Sie Interesse an dem Ergebnisbericht?

☐ Ja

☐ Nein

1. Möchten Sie an der Verlosung des Gewinnspiels als Dankeschön für Ihre Teilnahme mitmachen?

☐ Nein

☐ Ich möchte an der Verlosung zur Risikoeinstellung teilnehmen und bin

damit einverstanden, dass meine E-Mail-Adresse bis zur Ziehung der

Gewinner gespeichert wird. Meine Antworten der Befragung bleiben

weiterhin anonym, meine E-Mail-Adresse wird nicht an Dritte

weitergegeben.

☐ keine Antwort

47 . Wenn 46 ja: Bitte teilen Sie und ihre E-Mail Adresse….

_____________

..oder Postadresse mit:

_____________
